# Estimating Genetic Conformism of Korean Mulberry Cultivars Using Random Amplified Polymorphic DNA and Inter-Simple Sequence Repeat Profiling

**DOI:** 10.3390/plants7010021

**Published:** 2018-03-15

**Authors:** Sunirmal Sheet, Kuntal Ghosh, Satabdi Acharya, Kwang-Pyo Kim, Yang Soo Lee

**Affiliations:** 1 Department of Forest Science and Technology, College of Agriculture and Life Sciences, Chonbuk National University, 567 Baekje-daero, Deokjin-gu, Jeonju-si, Jeollabuk-do 561756, Korea; sunirmal123@jbnu.ac.kr or sunirmal.micr@gmail.com; 2 Department of Food Science and Technology, College of Agriculture and Life Sciences, Chonbuk National University, 567 Baekje-daero, Deokjin-gu, Jeonju-si, Jeollabuk-do 561756, Korea; micro.kuntal@jbnu.ac.kr (K.G.); kpkim@jbnu.ac.kr (K.-P.K.); 3 Department of Microbiology, Panskura Banamali College, Panskura, West Bengal 721152, India; satarya97@gmail.com

**Keywords:** mulberry cultivar, cluster analysis, genetic assortment, molecular markers, polymorphic index, ISSR, RAPD

## Abstract

Apart from being fed to silkworms in sericulture, the ecologically important Mulberry plant has been used for traditional medicine in Asian countries as well as in manufacturing wine, food, and beverages. Germplasm analysis among Mulberry cultivars originating from South Korea is crucial in the plant breeding program for cultivar development. Hence, the genetic deviations and relations among 8 *Morus alba* plants, and one *Morus lhou* plant, of different cultivars collected from South Korea were investigated using 10 random amplified polymorphic DNA (RAPD) and 10 inter-simple sequence repeat (ISSR) markers in the present study. The ISSR markers exhibited a higher polymorphism (63.42%) among mulberry genotypes in comparison to RAPD markers. Furthermore, the similarity coefficient was estimated for both markers and found to be varying between 0.183 and 0.814 for combined pooled data of ISSR and RAPD. The phenogram drawn using the UPGMA cluster method based on combined pooled data of RAPD and ISSR markers divided the nine mulberry genotypes into two divergent major groups and the two individual independent accessions. The distant relationship between Dae-Saug (SM1) and SangchonJo Sang Saeng (SM5) offers a possibility of utilizing them in mulberry cultivar improvement of *Morus* species of South Korea.

## 1. Introduction

The *Morus* species, commonly known as mulberry, belongs to the family of Moraceae and Urticales. The mulberry plants were reported as a truly wild species in the tropical and sub-tropical regions of the Himalayan terrain of India [1]. Recently, Vavilov et al. reported that the mulberry plants primarily originated from the China-Japan gene center, which includes Japan, Korea, and east China [2]. These plants are grown worldwide for different purposes. The plants have been used as an exemplary source of medicines and several drugs [3]. Moreover, mulberry fruits have applications in many industries, including the food industry. In South Korea, the plants are grown to feed silkworms (*Bombyx mori*) during the production of silk [4]. In Southern Europe, Asia, and the Southern United States, mulberry woody plants are used for handicrafts and landscaping. Breeding is necessary to develop varieties of mulberry plants with improved yields, quality, and tolerance to adverse environments, diseases, and pests. Therefore, more than thousands of *Morus* cultivars have been raised all over the world. The cultivars have been produced through intensive selection processes (e.g., hybridization, mutation breeding, or open pollination) or selective breeding processes (e.g., using vegetative stem cuttings) [4]. This breeding process has been followed to improve crop cultivars in terms of, e.g., resistance to biotic and abiotic stress, agronomical and quality characteristics, the genetic base of cultivars, and genetic variability [5]. Detailed genetic information about the parent species is needed to develop new varieties. 

DNA markers that are heritable, easy to evaluate, and not affected by the environment have been utilized to identify genetically divergent mulberry species. Among the DNA markers, random amplified polymorphic (RAPD) and inter-simple sequence repeat (ISSR) markers have been used often by several researchers to investigate genetic diversity between species. This is because this methodology is convenient and rapid [4,6]. The RAPD and ISSR rely on the usage of short PCR primers, which can bind to multiple sites in the plant genome. Moreover, the major advantage of both methods is that they do not require a priori knowledge about primer sequences in the target species [7]. Several researchers have used these techniques to identify the genetic diversity among different mulberry species [4,8,9]. In many population genetic studies, the RAPD and ISSR techniques have been frequently utilized in order to study the patterns of genetic diversity among Asian mulberry species [10,11]. The availability of different *Morus* species in South Korea was reported to be more than five hundred [5]. The common species of mulberry in South Korea are *M. alba* L., *M. indica* L., *M. bombycis Koidz*, *M. tiliaefolia Makino*, *M. nigra* L., and many other *Morus* species which are still unidentified [4,5]. For vegetative propagation, open pollination, and mutation breeding, many varieties of the cultivar are produced [4], and their genetic diversity is largely unexplored. Only Kalpana et al. (2012) has reported the high genetic similarities among the *Morus* species collected from various cultivars in the Jeonju region in the province of Chonbuk, South Korea, using RAPD and ISSR techniques [4]. Hence, the genetic diversity among the mulberry cultivar needs to be explored, as such knowledge may help to select varieties of plants with higher leaf and fruit yield potential. 

In the present study, we collected different mulberry cultivars from the Buan region in the Jeollabuk-do province of South Korea. Among them, only 1 cultivar (Dae-Suag) is cultivated for fruit production, whereas all of the others are cultivated for leaf productions (for either making tea or feeding silkworms). However, their genetic relationships have not been explored and remain unknown. Hence, we checked the genetic diversities among those cultivars using PCR-based RAPD and ISSR techniques.

## 2. Material and Methods

### 2.1. Plant Material Collection

The nine mulberry populations of different cultivars were obtained from the Seed Center for Agricultural Research and Extension Service of Buan, South Korea. The collected leaf samples were separately placed in air lock packs and stored at −80 °C for DNA extraction. The cultivars used in the present study and their morphological characteristics are listed in Table 1.

### 2.2. Genomic DNA Extraction 

The collected leaf samples were used to extract the genomic DNA. The extraction method was carried out using a Bio-Fact™ Genomic DNA Preparation Kit (Daejeon, Korea) and was done according to the manufacturer’s protocol. The quantification of isolated genomic DNA was performed on 0.8% agarose gels and the DNA was diluted to 10 ng/µL in order to obtain a uniform concentration for the following RAPD and ISSR analysis. 

### 2.3. RAPD Assay

A total of 10 RAPD primers (OPA and OPY) were selected on the basis of our previous work [4]. The primers are listed in Table 2. The PCR reaction volume of 30 µL contained 20 pmoles of primer, 2 U of *α*-Taq DNA polymerase (GeneAll, Seoul, Korea), 250 µM of dNTP (mixture), 20 ng of genomic DNA, 1× reaction buffer (2.5 mM of MgCl_2_), and 1× loading dye and stabilizer (GeneAll, Korea). The PCR amplification steps and their respective conditions were initial denaturation at 93 °C for 5 min, followed by 40 cycles of denaturation at 93 °C for 30 s, annealing at 36 °C for 30 s, elongation at 72 °C for 30 s, and the final extension at 72 °C for 15 min. The amplifications were carried out in an ESCO Swift Maxi^®^ Thermocycler (Horsham, PA, USA). The PCR amplification product was separated on 1.5% agarose gel using 1× TAE buffer for 2 h at 50 V. The agarose gels were stained with a 0.5 µg/mL ethidium bromide solution, and the gels were observed under a gel documentation system. 

### 2.4. ISSR Assay

Based on our previous work, a total of 10 ISSR primers with optimal reproducibility were used in the current study. The primers and their sequences are listed in Table 3. The amplification of ISSR primers was performed with 30 µL of a reaction mixture containing 1× reaction buffer (2.5 mM MgCl_2_), 250 µM dNTP (mixture), 20 pmoles of primer, 2 U of *α*-Taq DNA polymerase (GeneAll, Korea), 20 ng of genomic DNA, and 1× loading dye and stabilizer (GeneAll, Korea). The PCR amplification steps and their respective conditions were initial denaturation at 93 °C for 5 min, followed by 40 cycles of denaturation at 93 °C for 30 s, annealing at 60 °C for 30 s, elongation at 72 °C for 30 s, and the final extension at 72 °C for 15 min. After separation of the PCR product on 1.5% agarose gel, the agarose gels were stained with an ethidium bromide solution and documented under a gel documentation system. 

### 2.5. Marker Scoring and Statistical Analysis

The amplified DNA band fragments of the RAPD and ISSR assay were scored in binary characters, where 1 and 0 indicate the presence and absence of each band, respectively. Only bands repeatable in at least two independent experiments with the same primer set were used in the analysis. The reproducible bands were scored for analysis, and the absence of a band in at least one of different mulberry species was considered as polymorphic band. The polymorphism % was calculated from the following calculation:

Polymorphism % = (No of Polymorphic band/Total number of band) × 100.

The polymorphic information content (PIC) of each marker was calculated from the formula

PIC = 2*f*_i_ (1 − *f*i)

where *f*_i_ is the frequency of amplified allele (i.e., the present band), and (1 − *f*i) is the frequency of the null allele (i.e., the absent band) [12]. The genetic similarity coefficients among cultivars were calculated using the Nei and Li genetic similarity index formula,

SI = 2N_ij_/N_i_ + N_j_

where N_ij_ is the number of common bonds shared between i and j, and N_i_ and N_j_ are the total number of DNA bands for genotypes i and j, respectively [13]. Further, the matrix of presence or absence of a band is marked with 1, respectively 0, was used to compute the similarity matrix using Dice coefficient by software before cluster analysis [14]. Next, this similarity matrix was used to construct the phylogenetic tree through UPGMA (Unweighted Pair Group Method with Arithmetic Mean) method with a distance tool using the free PyElph 1.4 computer software [14,15,16]. The robustness of the dendrogram was further checked by the cophenetic correlation coefficient. The cophenetic correlation coefficient was calculated using the original genetic similarity matrix and the cophenetic matrix by the Mantel’s matrix correspondence test [17].

## 3. Results

In the present study, the results clearly define the interrelationships among the nine different mulberries collected from different cultivars of *Morus* in South Korea. To estimate the genetic variability, genomic DNA was isolated from leaf samples of all mulberries collected from different cultivars. The DNA integrity and quality were checked by running the DNA on agarose gel. The highly intact DNA was used for further assays. A total of 10 RAPD and 10 ISSR oligonucleotide primers were used to determine the diversity of mulberry cultivars in the current study.

### 3.1. Polymorphism Displayed by the RAPD and ISSR Markers

**Figure 1:**
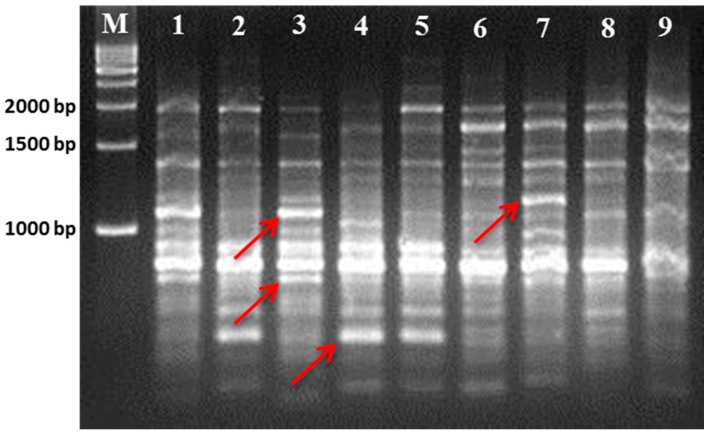
PCR amplification of DNA from nine mulberry genotypes with RAPD OPA-07 primer. The polymorphic bands are shown by an arrow symbol.

**Figure 2:**
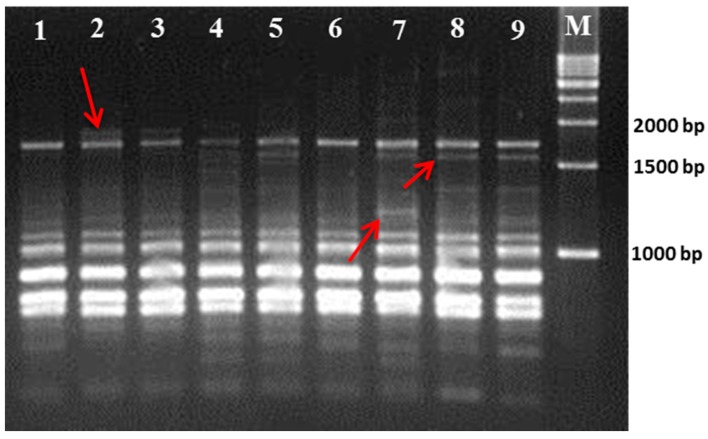
PCR amplification of DNA from nine mulberry genotypes with ISSR UBC-814 primer. The polymorphic bands are shown by an arrow symbol.

The results of the RAPD and ISSR analyses are summarized in Table 2 and Table 3, respectively. A total of 10 RAPD primers and 10 ISSR primers showed satisfactory polymorphism among the cultivars. All the primers produced reproducible amplified clear bands (as seen in Figure 1 and Figure 2). A total of 62 bands out of a total 104 amplified bands were polymorphic in nature for all the RAPD primers. The obtained PCR amplified product size range was >300–3000 bp for RAPD primers. In RAPD analysis, the polymorphism percentage was 59.62%. Moreover, the PIC value was calculated to find the ability of each RAPD primer marker to disclose polymorphic loci for each individual cultivar, and this is represented in Table 2. The OPY-07 primer showed the highest PIC value of 0.792, and the OPA-18 primer showed the lowest PIC value of 0.173 among all RAPD primers. The maximum number of amplified bands was obtained using the OPA-17 primer, where eight bands were polymorphic in nature. In ISSR analysis, a total of 82 bands was achieved, out of which 52 bands were found to be polymorphic. Thus, this produced 63.42% polymorphism. The UBC-820 primer exhibited the highest polymorphism (100%) and the highest PIC value (0.753) among the ISSR primers. The primer UBC-815 showed a maximum number of 12 bands, in which nine bands were polymorphic. In the ISSR assay, the amplified product size was observed to range from 150 to <2500 bp. 

### 3.2. Genetic Variance and Clustering Analysis between the Genotypes

**Figure 3:**
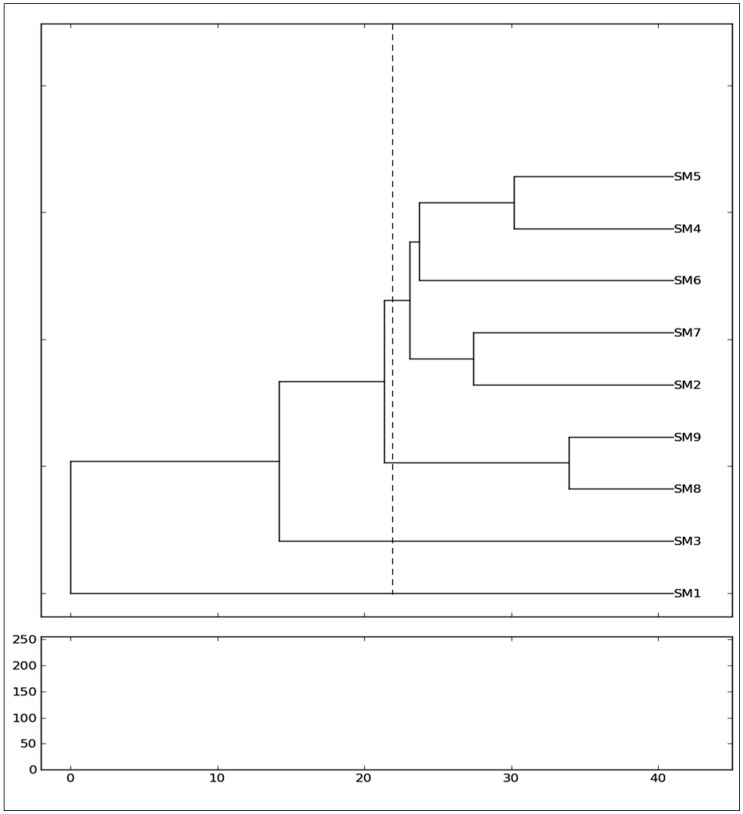
Dendrogram derived from UPGMA (unweighted pair group method using arithmetic average) clustering analysis of ISSR markers showing the genetic relationship among nine cultivars of mulberry. Genetic distances were labeled.

The intra-cultivar genetic similarity matrix was constructed on the basis of Nei and Li coefficient method. In the RAPD analysis, the genetic similarity coefficient of nine genotypes varied from a minimum of 0.361 between SM4 and Hong-Saug (SM9) to a maximum of 0.790 between Soo hyang (SM6) and Gwasang II (SM7) cultivars. There was an average similarity coefficient of 0.575 (Table 4). For ISSR markers, the genetic similarity coefficient ranged from 0.153 to 0.750 with an average coefficient of 0.451 (Table 5). A minimum similarity coefficient of 0.153 was calculated between the Dae-Saug (SM1) cultivar and Chung IL (SM2) cultivar, and a maximum coefficient of 0.727 was recorded between Gae-ryang (SM4) and SangchonJo Sang Saeng (SM5) cultivars. When the RAPD and ISSR data were pooled to construct a combined similarity matrix, the similarity coefficients varied from 0.183 to 0.814 (Table 6). The Dae-Saug and (SM1) was more distantly related to SangchonJo Sang Saeng (SM5), with a lower genetic similarity coefficient of 0.183. The Soosang was closely related to Hong-Saug with the high genetic similarity coefficient of 0.814. Later, the cluster analysis using the pooled data (RAPD and ISSR primers) and UPGMA simple agglomerative method grouped nine mulberry genotypes into two major clusters containing five and two mulberry genotypes (Figure 3). The SangchonJo Sang Saeng (SM5), Gae-ryang (SM4), Soo hyang (SM6), Gwasang II (SM7), and Chung IL (SM2) remained in one major group, whereas Soosang (SM8) and Hong-Saug (SM9) were clustered together in another group. Hong-ol (SM3) and Dae-Saug (SM1) genotypes stayed as an independent accession (Figure 3).

## 4. Discussion

Mulberry, an ecologically and economically important plant, has been cultivated by farmers as early as 5000 years ago [18]. This plant is still being cultivated around the world due to its industrial value, especially in sericulture and other purposes. Hence, improved mulberry varieties are necessary for mulberry crop improvement. The mulberry, belonging to the genus *Morus*, is a complicated species, containing many different and similar traits that vary depending on the cultivated region, environmental conditions, and developmental stages [19]. The conventional breeding process has been undertaken to breed mulberry cultivar during the past few decades in order to develop the mulberry varieties with high yield crops [5]. Thus, the evaluation of the genetic relationship of *Morus* genotypes is important for such tasks as measuring the genetic divergence among different mulberry cultivars, predicting progeny performance, and selecting parents for hybridization. 

Classifications and evolutionary relationships among *Morus* species have been determined by the phenotypic features of the plants, such as height form, leaf structure, bark structure, syncarp, fruit properties, flour morphology, and other various characters. The morphological features of the vegetative and reproductive structures vary from cultivar to cultivar [20], which is also evident from our study (Table 1). Compared to the seven other cultivars, Gae-ryang (SM4) and Soosang (SM8) have a small leaf surface. In contrast, the Sangchon Jo Sang Saeng (SM5) cultivar has large leaves. The Hong-ol (SM3) and Soosang (SM8) cultivars have lobed leaves, while the Gwasang II (SM7), Soo hyang (SM6), and Dae-Saug (SM1) cultivars have unlobed leaves. However, the disadvantages of phenotype-based classification are overcome by advanced molecular techniques, such as the use of microsatellite markers, which are highly neutral and insensitive to environment influence [21]. Hence, we used RAPD- and ISSR-based molecular markers to determine the genetic relationships among those cultivars. 

The results of the present study again suggest that RAPD and ISSR markers are useful in determining the intra-genetic relationship within mulberry genotypes. Although the ISSR markers in the present study exhibited a higher polymorphism in comparison to the RAPD markers, both markers produced almost satisfactory polymorphisms among the closely related genotypes. This finding resembles that of a previously published study on *Morus* species by Srivastava et al. (2004) [17], who showed that ISSR primers were more efficient in revealing DNA polymorphisms. Similarly, Vijayan and Chatterjee (2003) also found high genetic deviation among 11 closely related local cultivars of mulberry after ISSR markers were used [22]. PIC values have been estimated for both RAPD and ISSR markers, and the values have projected an estimate of the discriminating power of these markers [23]. The average PIC value using ISSR primers was found to be higher than those using RAPD primers. This outcome was in agreement with findings from Medhi et al. (2014), who found high PIC values via ISSR during an analysis of the genetic diversity of *Zanthoxylum* species [24]. Taken together with other reports [4], our findings further suggest that ISSR primers can be used as potential markers for discriminating inter-species of *Morus* cultivars.

Regarding the genetic relationship of the mulberry genotypes, the assessment of genetic similarity coefficients and the consequent clustering of the genotypes exposed a high genetic variability among the genotypes of nine *Morus* species, although they are collected from closely related local cultivars of the same origin. The correlation coefficient among matrices, based on Mantel’s test, was found to be high between RAPD and ISSR (*r* = 0.574, *p* = 0.000), RAPD and pooled combined matrices (*r =* 0.784, *p* = 0.001), and ISSR and pooled combined matrices (*r =* 0.74, *p* = 0.000). The dendrogram drawn using the UPGMA cluster method based on the combined pooled data of RAPD and ISSR markers divided the nine mulberry genotypes into two divergent major groups and the two individual independent accessions. The cophenetic correlation coefficient (*r* = 0.785) between the dendrogram and its original matrix, as verified by Mantel’s Z-statistics, was highly significant (*p* = 0.000). The grouping of five genotypes of *Morus alba* from different cultivars (SangchonJo Sang Saeng (SM5), Gae-ryang (SM4), Soo hyang (SM6), Gwasang II (SM7), and Chung IL (SM2)) as a major cluster revealed close genetic similarities among them (Figure 3), even though the genotypes have different morphological leaf characteristics (Table 1). Zhao et al. (2007) also reported that the same clustered genotypes contain differences even though they originated from the same ecotype [25]. It is a well-known fact that the sustainability of any plant population depends on their genetic diversity. Moreover, factors such as population isolation, genetic drift, mating system, gene flow, selection, and shifts in distribution are responsible for alterations in complex genetic constituting and further cause variations in hereditary multiplicity within the population [26,27]. The position of the genotype from Soo hyang (SM6) in relation to other genotypes merits special mention, as this genotype was found to have an intermediate genetic relationship between four other genotypes within the major cluster. The two genotypes of *Morus alba* collected from Soosang (SM8) and Hong-Saug (SM9) cultivars formed a separate group, which indicated its genetic divergence from the above genotypes (Figure 3). The separate independent position in the tree of *Morus alba* from Hong-ol (SM3) showed its greater genetic divergence from other *Morus alba* genotypes (Figure 3). Being a different species, the *Morus lhou* from Dae-Saug showed a separate identity which was observed in the phylogenetic tree. Thus, the present study showed the efficiency of unravelling the genetic correlation between closely related genotypes. The more closeness observed among *Morus alba* in a group that crosses among these individuals may not produce a substantial amount of heterosis. Hence, the cross-breeding within the cultivars in a group may not generate greater variety with higher seed fertility, and it will not be beneficial in transgressive breeding [17]. It is advisable that genetically diverse cultivars (e.g., *Morus alba* (SangchonJo Sang Saeng, (SM5)) and *Morus lhou* (SM1) or the cultivars from different clusters) can be interspecifically hybridize for a greater improvement of the mulberry cultivars in South Korea. Our study with the two markers endorse this view to a certain extent; however, considering the small number of genotypes and markers used for this analysis, a study with different types of markers and a higher number of genotypes needs to be undertaken. 

## 5. Conclusions

The present study represents an attempt to better understand the genetic assortment of *Morus* species collected from different cultivars of South Korea. Although there were high genetic similarities among the majority of mulberry cultivars, four distinct groups could be identified based on the RAPD and ISSR molecular markers. The segregation of the cultivars will further help in plant breeding (inter-group) for producing varieties with higher leaf and fruit quality. However, more cultivars need to be studied to determine the clear genetic diversity.

## Figures and Tables

**Table 1:** The selected mulberry cultivars used in the present study along with their morphological characters and region of origin. Leaf sizes are represented as (mean ± SD) of 10 representative samples.

Sample No	Species	Morphological Characters	Leaf Size (cm)		Cultivar
			Width	Length	
SM1	*M. lhou*	Leaf bluntly tipped, cros-venulate, margin serrate, rough surface, unlobed.	9.63 ± 3.12	12.04± 1.20	Dae-Saug
SM2	*M. alba*	Leaf ovate with serrate, often lobed, small smooth base.	10.33 ± 1.97	13.3 ± 0.57	Chung IL
SM3	*M. alba*	Leaf slightly cordate, margin serrate, lobed, comparatively smooth base, cros-venulate.	12.85 ± 0.27	15.3 ± 1.87	Hong-ol
SM4	*M. alba*	Leaf ovate with often lobed and serrate, cros-venulate, rough surface.	8.50 ± 2.42	10.3 ± 1.33	Gae-ryang
SM5	*M. alba*	Leaf bluntly tipped with small point, comparatively big rough surface, cros-venulate, unlobed, margin serrate.	16.20 ± 0.57	18.3 ± 1.22	SangchonJo Sang Saeng
SM6	*M. alba*	Elliptical leaf, unlobed, margin serrate, less rough surface, comparatively deep green, cros-venulate.	11.85 ± 2.27	12.87 ± 1.1	Soo hyang
SM7	*M. alba*	Elliptical leaf, unlobed, margin serrate, less rough surface, cros-venulate.	12.34 ± 1.46	15.3 ± 0.37	Gwasang II
SM8	*M. alba*	Leaf ovate with lobed and serrate, cros-venulate, rough surface.	9.42 ± 0.79	13.5 ± 2.04	Soosang
SM9	*M. alba*	Leaf slightly ovate with unlobed and serrate, cros-venulate, rough surface.	11.78 ± 1.70	13.4 ± 0.50	Hong-Saug

**Table 2:** Nucleotide sequences, the exhibited polymorphism percentage, and the polymorphic information content (PIC) of random amplified polymorphic DNA (RAPD) markers.

Primer Name	Sequence (5ʹ–3ʹ)	Polymorphic Band	Polymorphism (%)	PIC
OPA-02	TGCCGAGCTG	4	63.7	0.211
OPA-06	GGTCCCTGAC	10	90	0.335
OPA-07	GAAACGGGTG	7	71.82	0.415
OPA-14	TCTGTGCTGG	3	62.5	0.337
OPA-15	TTCCGAACCC	4	67.76	0.352
OPA-17	GACCGCTTGT	8	100	0.601
OPA-18	AGGTGACCGT	3	44.7	0.173
OPY-07	AGAGCCGTCA	9	100	0.792
OPY-15	AGTCGCCCTT	7	91.3	0.577
OPY-20	AGCCGTGGAA	7	100	0.775

**Table 3:** Nucleotide sequences, exhibited polymorphism percentage and PIC of inter-simple sequence repeat (ISSR) markers.

Primer Name	Sequence 5ʹ–3ʹ	Polymorphic Band	Polymorphism (%)	PIC
UBC-807	AGAGAGAGAGAGAGAGT	3	60	0.411
UBC-808	AGAGAGAGAGAGAGAGC	5	50	0.335
UBC-809	AGAGAGAGAGAGAGAGG	2	72.12	0.615
UBC-810	GAGAGAGAGAGAGAGAT	5	62.5	0.567
UBC-814	CTCTCTCTCTCTCTCTA	7	77.15	0.652
UBC-815	CTCTCTCTCTCTCTCTG	9	45.2	0.287
UBC-817	CACACACACACACACAA	6	70	0.603
UBC-820	GTGTGTGTGTGTGTGTC	3	100	0.753
UBC-824	TCTCTCTCTCTCTCTCG	5	80.2	0.632
UBC-825	ACACACACACACACACT	7	81.3	0.645

**Table 4:** Genetic similarity coefficient among nine mulberry cultivars obtained from RAPD markers.

Genotypes	SM1	SM2	SM3	SM4	SM5	SM6	SM7	SM8	SM9
SM1	0	-	-	-	-	-	-	-	-
SM2	0.733	0	-	-	-	-	-	-	-
SM3	0.780	0.714	0	-	-	-	-	-	-
SM4	0.620	0.647	0.685	0	-	-	-	-	-
SM5	0.650	0.669	0.714	0.566	0	-	-	-	-
SM6	0.714	0.658	0.833	0.700	0.727	0	-	-	-
SM7	0.685	0.633	0.738	0.737	0.633	0.790	0	-	-
SM8	0.766	0.620	0.662	0.592	0.620	0.678	0.727	0	-
SM9	0.452	0.460	0.592	0.361	0.564	0.614	0.589	0.483	0

**Table 5:** Genetic similarity coefficient among nine mulberry cultivars obtained from ISSR markers.

Genotypes	SM1	SM2	SM3	SM4	SM5	SM6	SM7	SM8	SM9
SM1	0	-	-	-	-	-	-	-	-
SM2	0.153	0	-	-	-	-	-	-	-
SM3	0.222	0.588	0	-	-	-	-	-	-
SM4	0.526	0.444	0.521	0	-	-	-	-	-
SM5	0.470	0.500	0.571	0.727	0	-	-	-	-
SM6	0.235	0.625	0.381	0.545	0.601	0	-	-	-
SM7	0.285	0.600	0.480	0.535	0.583	0.750	0	-	-
SM8	0.526	0.556	0.527	0.665	0.636	0.545	0.615	0	-
SM9	0.434	0.545	0.667	0.642	0.615	0.538	0.666	0.714	0

**Table 6:** Genetic similarity coefficient among nine mulberry cultivars obtained from pooled markers from RAPD and ISSR markers.

Genotypes	SM1	SM2	SM3	SM4	SM5	SM6	SM7	SM8	SM9
SM1	0	-	-	-	-	-	-	-	-
SM2	0.253	0	-	-	-	-	-	-	-
SM3	0.230	0.310	0	-	-	-	-	-	-
SM4	0.226	0.244	0.221	0	-	-	-	-	-
SM5	0.183	0.270	0.271	0.747	0	-	-	-	-
SM6	0.195	0.425	0.312	0.635	0.621	0	-	-	-
SM7	0.315	0.678	0.327	0.535	0.583	0.650	0	-	-
SM8	0.422	0.556	0.527	0.465	0.436	0.345	0.315	0	-
SM9	0.434	0.545	0.567	0.442	0.415	0.338	0.366	0.814	0
